# Scoring tail damage in pigs: an evaluation based on recordings at Swedish slaughterhouses

**DOI:** 10.1186/1751-0147-54-32

**Published:** 2012-05-28

**Authors:** Linda J Keeling, Anna Wallenbeck, Anne Larsen, Nils Holmgren

**Affiliations:** 1 Department of Animal Environment and Health, Swedish University of Agricultural Sciences, P.O. Box 7068, SE, 750 07, Uppsala, Sweden; 2 Swedish Animal Health Service, SE, 532 89, Skara, Sweden

**Keywords:** Swine, Animal welfare, Welfare assessment, Tail biting, Slaughter

## Abstract

**Background:**

There is increasing interest in recording tail damage in pigs at slaughter to identify problem farms for advisory purposes, but also for benchmarking within and between countries as part of systematic monitoring of animal welfare. However, it is difficult to draw conclusions when comparing prevalence’s between studies and countries partly due to differences in management (e.g. differences in tail docking and enrichment routines) and partly due to differences in the definition of tail damage.

**Methods:**

Tail damage and tail length was recorded for 15,068 pigs slaughtered during three and four consecutive days at two slaughterhouses in Sweden. Tail damage was visually scored according to a 6-point scale and tail length was both visually scored according to a 5-point scale and recorded as tail length in centimetres for pigs with injured or shortened tails.

**Results:**

The total prevalence of injury or shortening of the tail was 7.0% and 7.2% in slaughterhouse A and B, respectively. When only considering pigs with half or less of the tail left, these percentages were 1.5% and 1.9%, which is in line with the prevalence estimated from the routine recordings at slaughter in Sweden. A higher percentage of males had injured and/or shortened tails, and males had more severely bitten tails than females.

**Conclusions:**

While the current method to record tail damage in Sweden was found to be reliable as a method to identify problem farms, it clearly underestimates the actual prevalence of tail damage. For monitoring and benchmarking purposes, both in Sweden and internationally, we propose that a three graded scale including both old and new tail damage would be more appropriate. The scale consists of one class for no tail damage, one for mild tail damage (injured or shortened tail with more than half of the tail remaining) and one for severe tail damage (half or less of the tail remaining).

## Background

Tail biting can be described as the chewing and biting of another pig’s tail. Besides pain from acute injuries on the damaged tail, receivers often suffer from secondary infections leading to abscesses. Consequently, carcasses from tail bitten pigs are in many cases partly or fully condemned at slaughter [1-3]. Thus in addition to being a welfare problem for the bitten pig, tail biting is also an economic problem for the farmer. When large numbers of animals are considered in a representative sample, the prevalence of tail biting can also be a reflection of the housing systems and management practices in a region or country and hence an indicator of the welfare of the pigs in general.

Tail damage is routinely recorded at slaughter in some countries. In commercial pig production e.g. in Sweden and Norway; these recordings are continuously used as an indication of tail biting at farm and regional level. In Sweden monthly averages for each slaughterhouse are saved in a database as described by Lundeheim et al. [4]. Tail damage has been reported to vary between 1% and 3% among pigs in Sweden [3,5] and around 4% of the pigs slaughtered in Norway have tail damage [6]. Tail docking is banned in both Sweden and Norway. Estimates of tail damage prevalence from most other countries are based on specific studies. In a survey of 62,971 pigs involving 6 slaughterhouses in the United Kingdom, Hunter et al. [7] found that 9% of undocked pigs and 3% of docked pigs had damaged tails. Moreover, they found that 0.1% of docked and 0.5% of undocked pigs had the most severe form of damage (i.e. parts of the tail missing and severe wounds with swelling and signs of infections). Valros et al. [8] found the total prevalence of tail damage (any type; from scratches on the skin to severe wounds and shortened tails) in 10,852 pigs recorded at a single slaughterhouse in Finland to be 34.6% and that the prevalence of fresh tail lesions and severe damage was 11.7% and 1.3% respectively.

It is difficult to draw conclusions when comparing prevalence’s between studies and countries. One reason is differences in management, especially regarding tail docking and enrichment, prescribed by law in different countries. Another reason is the difference in the definition of tail damage. The definition used in routine classification of tail damage in Sweden is that “at least half of the tail is missing or clear signs of bite damage on the tail” [9].

Taking the definition used in routine classification of tail damage in Sweden and findings in other countries, such as the high prevalence reported from Finland by Valros et al. [8], into account, it could be suspected that the true prevalence of tail damage in Sweden is considerably higher than reported from the routine recordings. This underlines the difficulty of comparing results from different investigations, and the need for a standardized scoring and recording system for tail damage.

The aim of this study was to assess the prevalence and severity of tail damage in a representative sample of pigs at slaughter in two slaughterhouses located in different regions of Sweden, to compare the scientifically recorded tail damage prevalence with routine slaughterhouse recordings and to propose a feasible scoring system that could be used for benchmarking nationally and internationally.

## Methods

Tail injury, tail length and sex were recorded for all pigs slaughtered over four days (16–19 June, 2003) at slaughterhouse A (western Sweden) and over three days (19–21 August, 2003) at slaughterhouse B (southern Sweden). The recording period of three or four days was chosen to maximise the number of pigs scored, but reduce the risk that the sample was dominated by pigs from only a few farms.

The person performing the recordings in the slaughterhouses was positioned after bleeding and the first wash, but before the thorough wash and scalding of the carcasses on the slaughter line. This position was chosen due to the high frequency of mechanical damages to the tail seen after the second washing and because the person was able to be close enough to the line to touch and examine the tails closely. The observations were carried out by six people; four in slaughterhouse A and four in slaughterhouse B, with two people recording in both slaughterhouse A and B. All observers recorded a representative proportion of male and female pigs because of the long duration of observation bouts at the slaughter line.

Tail injury was judged visually and scored according to a 6-point scale; 0 = no injury, 1 = swollen, 2 = small sore or wound, 3 = small sore or wound and swollen, 4 = major sore or wound, 5 = major sore or wound and swollen. Both fresh and older wounds were scored. The distinction between a small and major sore or wound was agreed upon between the observers in preliminary visits to the slaughterhouse and was based upon a combination of size and depth. Tail length was judged visually and scored according to a 5-point scale; 0 = full length, 1 = greater than 75% remaining, 2 = between 50 and 75%, 3 = between 25 and 50%, 4 = less than 25% of tail remaining. Tail length was additionally recorded as tail length in centimetres for pigs with injured or shortened tails.

Tail injury was analysed according to the full 6-point scale and according to a 2-point scale including ´no injury’ (score 0–2) and ‘injury’ (score 3–5). Only 1.7% of the pigs were scored as having score 1 or 2 on the 6-point scale and 76% of these (i.e., 1.3% of all pigs) were recorded at slaughterhouse A. This difference was a consequence of a discussion among observers of whether or not a specific type of small sore at the very tip of the tail, resulting in a tail damage score 2 in slaughterhouse B. These sores are described by Nørregård et al. [10]. The decision that it was not, led to fewer pigs being given tail damage score 2 in slaughterhouse B. Therefore, to make a fair comparison between slaughterhouses and observers, scores 1 and 2 were both considered as ‘no injury’ in the analysis on the 2-point tail biting injury scale. Tail length was also analysed in two ways; according to the full 5-point scale and according to the 2-point scale ‘more than half of the tail remaining’ (score 0–2) and ‘half or less of the tail remaining’ (score 3–4).

The statistical analyses were performed using SAS version 9.2 (SAS Institute Inc. Cary, NC, USA). Data were analysed with descriptive statistics (procedure FREQ), Pearson correlation for agreement between scores for tail length and length in centimetres (procedure CORR), general linear models for length of tail in centimetres (procedure GLM, normality tested with procedure UNIVARIATE), generalised linear models with cumulative logit link and multinomial distribution for full scale tail injury- and tail length scores (procedure GENMOD) and generalised linear models with logit link and binary distribution for the 2-point scales of tail injury and tail length scores (procedure LOGISTIC). All statistical models included the fixed effects of slaughterhouse, sex of the pig and observer nested within slaughterhouse. The effect of slaughterhouse was excluded from the statistical model when analyses were performed for each slaughterhouse separately.

## Results

A total of 15,068 pigs, 6,837 and 8,231 pigs in slaughterhouse A and B respectively, were scored. Of these, 50.8% were male in slaughterhouse A and 50.7% in slaughterhouse B. The total prevalence of any type of injury and/or shortening of the tail was 7.0% (8.5% males; 5.5% females) in slaughterhouse A and 7.2% (9.1% males; 5.2% females) in slaughterhouse B. In slaughterhouse A, there was a small difference (0.7%) in the frequency of pigs with injured and/or shortened tails if all injury scores on the scale were used (scores 1–5) compared to when only major injury (scores 3–5) was used. However, there was no difference in frequency of injured pigs depending on the scale used in slaughterhouse B. If only pigs with half or less of the tail remaining were considered, the percentages were 1.5% and 1.9% slaughterhouse A and B respectively (Table 1). According to the slaughterhouse’s own routine recordings, monthly averages corresponding to the time of the data collection in the present study were 1.2% and 1.6% at slaughterhouses A and B respectively.

**Table 1:** Total number and percentage of pigs slaughtered in slaughterhouse A and slaughterhouse B with injured and shortened tails

	Slaughterhouse A	Slaughterhouse B
Total number pigs	6837	8231
*Injured (score 1-5) and/or shortened tail (%)*		
All pigs	7.0	7.2
Males	8.5	9.1
Females	5.5	5.2
*Major injury (score 3-5) and/or shorter tail (%)*		
All pigs	6.3	7.2
Males	7.8	9.1
Females	4.7	5.2
*Less than half tail remaining (%)*		
All pigs	1.5	1.9

### Tail length score and measure in centimetres

The visually judged tail length score applied in this study was significantly correlated with the tail length measured in centimetres (r = −0.94, P < 0.001, N = 1053).

### Differences between sexes

A higher percentage of males had injured and/or shortened tails compared with females (8.8% vs. 5.3%, P < 0.001). Males tended to be more severely bitten than females when the full 6-point scale was analysed (P = 0.07) and significantly when the simpler 2-point scale including ‘no injury’ and ‘injury’ was analysed (P = 0.02).

### Differences between slaughterhouses

There was no significant difference between slaughterhouses in frequency of pigs with tail injury when the full 6-point scale was analysed (P = 0.16) nor when the 2-point scale including ‘no injury’ and ‘injury’ was analysed (P = 0.92). There was a significant difference between slaughterhouses in tail length according to the 5-point tail length scale (P < 0.001) indicating longer tails in slaughterhouse B, but this difference was not found using the 2-point scale ‘more than half of the tail remaining’ and ‘half or less of the tail remaining’ (P = 0.88).

### Differences between observers

Comparisons were made of the percentage of different injury and tail length scores recorded by the different observers. There was an overall significant difference between observers in the percentages of the different injury scores given (P < 0.001), as well as differences between observers at slaughterhouse A (P <0.001), but not between observers at slaughterhouse B (P = 0.90). Further analysis showed that the main difference between observers was associated with differences in classification score ‘2’ for tail injury. On the 2-point scale including ‘no injury’ and ‘injury’, there were no overall differences between observers (P = 0.91) or between observers at slaughterhouse A (P = 0.88) nor at slaughterhouse B (P = 0.64). There was an overall significant difference between observers in the percentages of the different tail length scores given (P < 0.001) as well as differences between observers in tail length measured in centimetres (P <0.001). This difference was also seen when the 5-point scale was transformed into a 2-point scale including ‘more than half of the tail remaining’ and ‘half or less of the tail remaining’ (P = 0.01). There were significant differences between observers for both tail length measures (score and length in cm) also within slaughterhouse, e.g. the effect of observer on the 2-point scale for tail length at slaughterhouse A (P = 0.05) and at slaughterhouse B (P = 0.01). It was because of these inter observer differences that observer was always adjusted for in the statistical model.

## Discussion

The number of pigs with tail damage (injury or shortened tails) at slaughter was found to be considerably higher than indicated by the remarks from routine recordings from the same time period. According to the classification used in this study, around 7% of the pigs had some form of tail damage. When only cases with half or less of the tail remaining were considered in the current study, the percentage of pigs (between 1-2%) was in agreement with the monthly averages of tail damaged pigs per slaughterhouse, despite some evidence of inter observer variability in the scoring of tail length. This finding suggests that the routine recordings of tail damage at slaughter in Sweden reflects the number of pigs with half or more of the tail missing rather than pigs with damaged, but longer tails. Thus they leave a considerable amount of tail damage unseen in the reported statistics from slaughterhouses.

The current method of routine slaughterhouse recording is adequate for flagging farms with major tail biting problems, so that they can be targeted by the advisory services. However, the results from the present study indicate that the routine method, as it is currently practiced, misses the majority of tail damaged pigs and is therefore inadequate for benchmarking purposes. If all visible tail damage is taken into consideration, the true prevalence in Sweden is more than four times higher than the routine recordings at slaughter indicate. If the two graded scale implemented at the slaughterhouse is not sensitive enough to reflect the true prevalence for benchmarking purposes, the question arises of what scale would be most appropriate.

The Welfare Quality® protocol includes a 3-point scale including a) ‘No evidence of tail biting’, b) ‘Indication of superficial biting along the length of the tail, but no evidence of fresh blood or of any swelling (red areas in the tail are not considered as wounds unless associated with fresh blood)’ c) fresh blood is visible on the tail; there is evidence of some swelling and infection; part of the tail tissue is missing and a crust has formed”, for on-farm assessment of tail biting on individual pigs. Thus the emphasis is on on-going outbreaks, with fresh wounds, rather than on identifying a previous outbreak. For the classification on herd level, the Welfare Quality® protocol uses a 2-point scale including one class for percentage of pigs with no tail biting (corresponding to levels ‘a’ and ‘b’ in the 3-point scale) and one class for tail biting (corresponding to level ‘c’ in the 3-point scale) [11][12]. Thus we propose that the Welfare Quality’s herd level classification would underestimate the true prevalence of tail damage in the same way as the present study found the Swedish routine recordings at slaughter underestimates them.

The Welfare Quality® system includes a protocol for recording of tail damage on farm but no protocol for recording at the slaughterhouse.

**Figure 1:**
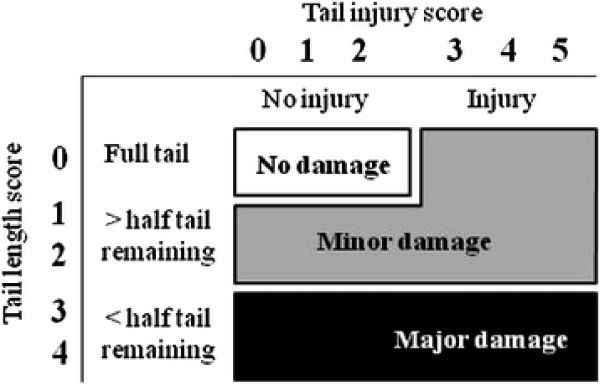
**Suggested tail damage scale classes (no, minor and major damage) for benchmarking purposes in practice, based on tail length and injury scores used in research.** If pigs have more than half of the tail docked, using only the tail injury score (no injury and injury) could be considered. This would have the effect of no longer distinguishing between minor and major damage, but would still allow comparisons with non-tail docked pigs in the percentage of pigs with no damage and therefore still be useful for benchmarking internationally.

Despite training of observers in the present study, the more detailed scoring of tail injury (6-point scale) and tail length (5-point scale) was not reliable when different observers were involved. This finding, together with the fact that there is a variation in routine recording between slaughterhouses [3] emphasizes the importance of a simple scoring system for routine slaughterhouse coding. To include the aspects of both underestimation of prevalence and observer reliability in a scale of tail damage at slaughter for benchmarking purposes, we suggest a three graded scale where both tail injury and tail length judgments evaluated in this study are included; one class for no tail damage, one intermediate class for mild tail damage, i.e. injured and/or shortened tail with more than half of the tail remaining, and one class for severe tail damage and/or less than half of the tail remaining (Figure 1). This scale combines information on the presence of new or old wounds on the tail by incorporating information on tail length. In this way all injuries, even completely healed ones, are included. Although we developed this scale at a slaughterhouse, the scoring system could also be used on-farm. If this scale is to be used in future, pictures illustrating the different scores would need to be agreed upon. Indeed if pictures had been used in this study, the slight difference in scoring between observers may have been reduced.

The average total tail damage in our study is considerably lower than the corresponding prevalence in Finland, [8], although this may be in part explained by the fact that the ban on tail docking in Finland may not have been fully implemented at that time. The level of tail damage seen in the present study is however in accordance with the prevalence that Hunter et al. [7] reported among undocked pigs in UK. Clearly though, tail docking will have contributed to the difference in tail damage prevalence in our Swedish study and studies where pigs were tail docked, for example in Denmark [13] and Northern Ireland [1]. Despite the growing amount of research and slaughterhouse data on tail damage, meta analyses to compare risk factors or track trends over time is difficult, in part because of differing methodologies and in part because some countries dock tails and others do not. Thus any scoring system at least has to be able to deal with docking of more than half the tail, unless this docking itself is regarded as a major damage. We propose that when tails are docked, only the injury scoring part of the scale is used, whereas both the injury and tail length scoring parts are used on non-tail docked pigs. In cases of tail damage assessments including both pigs with intact tails and pigs with docked tails, information about the practice of tail docking at pig level, or at least herd level (i.e. if practiced and, if so, the proportion of tail removed) should be included in the analyses. This would allow all possible comparisons between herds with docked and non-tail docked pigs, but with the most sensitive comparison (between non-tail docked pigs) being the one that is most likely to increase in the future if the current trend towards less docking of pigs, at least within the EU, continues.

In this study, the inconsistency in scoring between observers was mainly attributable to differences over one class, score 2, ‘small sore or wound’, and this inconsistency may have been exacerbated by discussions around a different type of tail damage, not caused by tail biting. As a consequence of these discussions, some tails were removed and injuries were studied in more detail in a supplementary study [10]. The inflammation and thickening showed similarities to tail tip necrosis seen in fattening bulls [14,15]. This might be supported by the fact that these tails were often coated in dry manure implying that the changes might be related to the hygiene level in the pen. Tail tip necrosis in fattening pigs should not be confused with tail necrosis in piglets, which is thought to be caused by environmental factors in the nursing pen [16].

This study yet again supports previous findings that males are more likely to have damaged tails than females [13,17,18]. Moreover, this study now also adds that among pigs with tail damage, the damage on the male pigs is more likely to be severe. This is in keeping with the results of Brunberg et al. [19] where females were found to perform a higher proportion of severe tail bites and tended to perform more severe tail bites than males. More vigorous biting behaviour by females may lead them to cause more tail damage.

## Conclusions

Tail damage recorded routinely at slaughterhouses in Sweden, as an indication of tail biting and hence general pig welfare, underestimates the actual prevalence. More detailed observations estimated the prevalence to be four times higher and our findings suggest that this is because, in practice, it is mainly cases with half or less of the tail remaining that are recorded. In specific studies or in animal welfare assessment systems for benchmarking purposes nationally or internationally, we suggest a three graded scale with one class for no tail damage, one intermediate class for minor tail damage i.e. either injured with no shortening of the tail or injured with some shortening of the tail but still with more than half of the tail remaining, and one class for major tail damage i.e. with less than half of the tail remaining irrespective of whether or not the tail has any apparent injury. This study supports that tail damage is more common in males than in females, but also adds that the damage is more severe in males than females.

## Competing interest

Although one author (NH) was employed by the national organisation that, among other things, advises the pig sector, none of the authors of this paper has a financial or personal relationship with other people or organisations that could inappropriately influence or bias the concept of the paper.

## Authors' contributions

LK, AL and NH planned the design of the study and carried out the practical data collection. AW carried out the statistical analyses. LK and AW drafted the manuscript and all authors read and approved the manuscript.
